# Attention-dependent modulation of neural activity in primary sensorimotor cortex

**DOI:** 10.1002/brb3.114

**Published:** 2013-01-04

**Authors:** Annette Milnik, Isabella Nowak, Notger G Müller

**Affiliations:** 1 Department of Neurology, University of MagdeburgMagdeburg, Germany; 2 Cognitive Neurology Unit and Brain Imaging Center, Clinic for Neurology, Johann Wolfgang Goethe-UniversityFrankfurt, Germany; 3 German Center for Neurodegenerative DiseasesMagdeburg, Germany

**Keywords:** Attention, distraction, dual task, finger tapping, functional MRI, hand area, motor task, primary motor cortex

## Abstract

Although motor tasks at most times do not require much attention, there are findings that attention can alter neuronal activity not only in higher motor areas but also within the primary sensorimotor cortex. However, these findings are equivocal as attention effects were investigated only in either the dominant or the nondominant hand; attention was operationalized either as concentration (i.e., attention directed to motor task) or as distraction (i.e., attention directed away from motor task), the complexity of motor tasks varied and almost no left-handers were studied. Therefore, in this study, both right- and left-handers were investigated with an externally paced button press task in which subjects typed with the index finger of the dominant, nondominant, or both hands. We introduced four different attention levels: attention-modulation-free, distraction (counting backward), concentration on the moving finger, and divided concentration during bimanual movement. We found that distraction reduced neuronal activity in both contra- and ipsilateral primary sensorimotor cortex when the nondominant hand was tapping in both handedness groups. At the same time, distraction activated the dorsal frontoparietal attention network and deactivated the ventral default network. We conclude that difficulty and training status of both the motor and cognitive task, as well as usage of the dominant versus the nondominant hand, are crucial for the presence and magnitude of attention effects on sensorimotor cortex activity. In the case of a very simple button press task, attention modulation is seen for the nondominant hand under distraction and in both handedness groups.

## Introduction

Usually, our motor system operates rather independently without the need to pay attention to the executed movements and daily life illustrates that within a multitasking situation, a trained motor task can be performed without devoting attention to it (e.g., driving a car while talking). In fact, with an overlearned motor task, giving attention to the task can even disturb its execution (e.g., Baumeister 1984). On the other hand, during learning of new motor sequences, distraction can decrease performance (Passingham 1996). After learning has taken place, explicit knowledge about what our motor system is doing diminishes. For example, when learning to type with 10 fingers, at the beginning one needs explicit knowledge of the exact keyboard position of each letter. After getting the routine, this knowledge is gradually lost. On a neurophysiological level, research has shown that attention to motor action entails neuronal activity changes in the premotor cortex, in prefrontal regions, and in mainly the left-parietal cortex (Jueptner et al. 1997; Rushworth et al. 2001; Rowe et al. 2002a,b). Regarding the primary motor cortex, it was observed that during learning of a new task attention to an external focus (button to be pressed) in comparison with an internal focus (moving finger) is associated with higher activity in this brain region (Zentgraf et al. 2009); this finding is paralleled by better task performance (Wulf and Prinz 2001; Wulf et al. 2010).

The primary motor cortex is not a homogenous entity but is divided into at least two anatomical, neurochemical, and functional distinct subregions, called 4a for the more anterior, lateral, and superior part and 4p for the more posterior, medial, and inferior part (Zilles et al. 1995; Geyer et al. 1996). Findings in monkeys also point to a dichotomy of the primary motor cortex (Stepniewska et al. 1993). Regarding attention modulation, Binkofski et al. (2002) observed that in right-handers, who performed a paced U-shaped movement with their right index finger, area 4p but not 4a was modulated by attention to action: directing attention to the moving finger led to more activity in 4p of the contralateral hemisphere; the regions of interest (ROIs) were defined anatomically. Johansen-Berg and Matthews (2002) investigated right-handers who used their left hand in a paced button press task, and demonstrated that simultaneous distraction by a cognitive task (counting backward) led to a decrease of activity in primary motor cortex of the contralateral hemisphere; this effect was more pronounced in area 4p than 4a, and the ROIs were defined anatomically. Rodríguez et al. (2004) showed a decrease of activity in the contralateral primary motor cortex during a phasic finger movement of the dominant hand under distraction; subjects were right- (*n* = 8) and left-handers (*n* = 2) and the ROIs were defined functionally. Rowe et al. (2002a) in turn reported no influence of attention, namely concentration on the moving finger, on primary motor cortex when investigating right-handers who did a paced sequential finger movement of the right hand; analysis was done on a whole-brain level. It is noteworthy that taken all studies together, only two left-handers were investigated (Rodríguez et al. 2004).

In summary, although previous studies suggest that attention can have some influence on primary motor cortex activity, the exact nature of these effects needs to be explored further. Factors like handedness, usage of the dominant versus nondominant hand, type of attention modulation (distraction vs. concentration), and hemisphere (contra- vs. ipsilateral motor cortex) need to be accounted for. Hence, in this study, we investigated both, right- and left-handers, when they moved the dominant, the nondominant, or both hands under four different attention conditions: attention-modulation free (tapping without further instruction), distraction (counting backward in steps of three while tapping), concentration (attention to the moving finger[s]), and divided concentration (concentration on only one of the fingers during bimanual movement). As movement frequency, task complexity, and motor learning status are known to influence primary motor cortex activity (Boecker et al. 1998; Jäncke et al. 1998; Toni et al. 1998; Debaere et al. 2004; Puttemans et al. 2005), we controlled for these factors by using a simple externally paced and controlled button press task with auditory cues. We used a simple externally paced button press task in order to avoid attention-related effects on task performance, as any behavioral difference would have confounded our interpretations of the observed neuronal activity in motor cortex. If, for example, distraction had caused a slowing in tapping, a reduction in motor cortex activity could have been simply attributed to the less frequent button presses instead of reflecting top-down modulation. By investigating both the dominant and the nondominant hand within the same individual, we were able to address whether attention-related modulations of primary motor cortex activity depend on the efficiency of the neural representations of the moving hand which we assume to be higher in the motor cortex of the dominant hand. Moreover, as we not only investigated right-handers but also left-handers, we were able to assess whether the postulated effects can be replicated in this group and hence generalize to the whole population.

As expected effect sizes were medium to small, we used a functional ROI-based approach. We divided the hand area of both hemispheres in two distinct subregions in order to assess whether the more posterior, medial, and inferior part (area 4p) is differentially influenced by attention in comparison with the more anterior, lateral, and superior part (area 4a). In order to assess whether our attention-related task modulations induced the expected activity changes in the attention network of the dorsal frontoparietal cortex (Collette et al. 2005; Fox et al. 2005; Nebel et al. 2005) on one hand and in the default network in the ventral frontotemporal cortex (McKiernan et al. 2003; Fox et al. 2005) on the other hand, we complemented our ROI analyses with a whole-brain analysis.

To summarize, our main hypotheses were the following: we expected (1) activity of the primary motor cortex to be reduced under distraction due to shared resources in the case of a concurring cognitive task; (2) enhanced activity of the primary motor cortex under concentration, reflecting attention-mediated top-down control; (3) attention effects to be more pronounced for movements with the nondominant hand in comparison with the dominant hand, due to less efficient network specification for the less often used nondominant hand; (4) in the case of unimanual movements larger effects of attention in the contralateral in comparison with the ipsilateral hemisphere, due to a higher task-specific activation; (5) a more pronounced effect in 4a in comparison with 4p; (6) none or at the most subtle behavioral effects due to the setting with an externally paced simple finger-tapping task; and (7) an influence of attention modulation (concentration and distraction) on the activity of the dorsal frontoparietal attention network.

## Materials and Methods

### Participants

Nineteen right-handed (mean age 24.7, range 20–34 years; seven men) and eight left-handed (mean age 27.9, range 20–51 years; one man) healthy subjects participated in the study. All subjects received a magnetic resonance imaging (MRI) safety screening and gave written consent. They were moderately financially rewarded for their participation in the study conducted in conformity with the declaration of Helsinki and approved by the local ethics committee.

### Design

We manipulated moving finger (index finger of dominant hand, of nondominant hand, of both hands) and attention type (“attention-modulation free” as cued tapping with no further instruction, “distraction” as cued tapping while counting backward, and “concentration” as cued tapping while actively paying attention to the moving finger[s]). In the bimanual task, there was an additional condition “divided concentration” defined as paying attention to either the moving index finger of the dominant or nondominant hand. In sum, there were 11 conditions that were assessed in an functional (fMRI) block design (see Table 1).

**Table 1:** Experimental tasks

Condition	Attention level	Index finger movement
1	Attention-modulation free	Dominant hand
2	Attention-modulation free	Nondominant hand
3	Attention-modulation free	Both hands
4	Distraction	Dominant hand
5	Distraction	Nondominant hand
6	Distraction	Both hands
7	Concentration	Dominant hand
8	Concentration	Nondominant hand
9	Concentration	Both hands
10	Divided concentration on dominant hand	Both hands
11	Divided concentration on nondominant hand	Both hands

**Figure 1:**
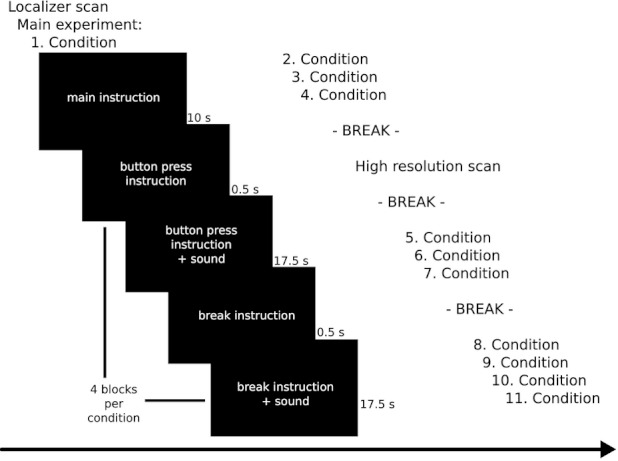
Experimental setup. Time course of the localizer scans, the 11 experimental conditions of the main experiment and the anatomical scan. Each experimental condition was repeated consecutively four times. Sequence of the 11 experimental conditions was assigned randomly. Before the 11 trials, all but three participants got the localizer scans (moving with right and left index finger alternating two times) for functional localization of the hand area.

Each of the 11 experimental conditions was presented in four blocks separated by blocks of rest (see Fig. 1). The sequence of the 11 experimental conditions was randomized. Handedness (see below) was used to assign dominant and nondominant hand as well as dominant and nondominant hemisphere for each subject. Movement frequency (main tapping frequency ascertained by fast Fourier transformation of the time series of button presses) and mean standard deviation of button presses in comparison to sound occurrence were determined as behavioral control variables.

### Procedure and stimuli

Participants received pretraining outside of the scanner consisting of a shortened version of the 11 tasks to make sure that they had understood the instructions. If they had any difficulties with any of the tasks, pretraining was repeated. Handedness was measured by the Edinburgh Handedness Inventory (Oldfield 1971). Three subjects revealed possible mixed-handedness (laterality quotient −50 to 50). In their cases, the writing hand was crucial for assigning handedness (two right-handers, one left-hander). Mean handedness laterality quotient was 87.15 for the right-hander group and −66.75 for the left-hander group. Fifty percent of the left-handers and 17% of the right-handers reported a family history of left-handedness.

Inside the scanner, instructions were projected with a beamer (Sony Data Beamers Type VPL-XP20, 1400 ANSI, Berlin, Germany) on a screen, which could be seen via a mirror mounted at the head coil. Sound was presented via MR compatible earphones (MR Confon, Magdeburg, Germany), and button presses were recorded with a custom-made fiberglass response box placed over the participant's belly to be usable with the right and left index finger. Stimuli and response recording were controlled by Presentation 9.9 software (Neurobehavioral Systems™, Albany, CA).

At the beginning of each experimental condition, the main instruction was presented for 10 sec: (1) “Press button with right (or left or both) index finger(s), as soon as sound begins,” (2) “Press button with right (or left of both) index finger(s), at the same time count silently backward from the appearing number in steps of three, as soon as sound begins,” (3) “Press button with right (or left or both) index finger(s), at the same time concentrate on the moving finger(s), as soon as sound begins,” or (4) “Press button with both fingers and concentrate on the right (or left) index finger, as soon as sound begins.” In order to signal to the subjects that the motor task was about to start, at the beginning of each block, a shortened button press instruction was presented for 0.5 sec without auditory cue. During the following 17.5 sec, 35 auditory cues were delivered every 0.5 sec (Presentation 9.9: channel 1 = 0.5 × sin [1000, 0, 200, 0], 2 Hz) while typing instruction was present. In the distraction condition, the appearing number was generated by chance as a number between 100 and 199 for each block separately. In every block, the active phase was always followed by a resting phase, whereby the resting instruction (“Break”) was shown in the initial 0.5 sec without any sound, followed by 17.5 sec of sound presentation, during which no button presses were required. Each of the 11 experimental conditions was repeated four times, so that each condition comprised 140 trials (button presses) presented in one experimental session with four blocks (for experimental setup see Fig. 1).

### Localizer session

At the beginning of the scanning session, participants performed a run of four conditions during which they had to alternately move the right and left index finger for functional localization of the associated subareas within the primary sensorimotor cortex. Instruction and course of events were the same as in the attention-modulation free, one-finger conditions of the main experiment. For one left-handed and two right-handed participants, no localizer scan was available. Hence, the attention-free, single finger-tapping blocks of the main experiments were used to map the hand areas for these subjects.

### MRI data acquisition

MRI data were acquired with a 3.0 Tesla MRI system (Allegra, Siemens, Erlangen, Germany) at the Brain Imaging Center in Frankfurt/Main, Germany. Functional images were obtained by using a T*-weighted transversal gradient-echo echo-planar image (EPI) sequence (repetition time 2000 msec, echo time 30 msec, flip angle 77°, 36 slices, slice thickness 3 mm, matrix 192 × 192 mm, gap 10%, in plane resolution 3.0 × 3.0 mm). In sum, 32 (4 × 8) fMRI volumes were collected per condition and subject. Three-dimensional high-resolution structural images were acquired using a T1-weighted sagittal gradient-echo (MP-RAGE) sequence (TR 2250 msec, TE 4.38 msec, flip angel 8°, inversion time T1 900 msec, 160 slices, slice thickness 1 mm, matrix 256 × 256 mm, gap 50%, in plane resolution 1.0 × 1.0 mm).

### Data analysis

#### Preprocessing fMRI data

Functional MRI data were preprocessed with Brainvoyager QX 1.7 (BrainInnovation, Maastricht, the Netherlands) software. Preprocessing involved slice scan time correction (sinc interpolation), 3D motion correction (trilinear interpolation), and temporal filtering (linear trend removal, high-pass filter three cycles in time course). The first five volumes of each functional run were discarded because of unsteady magnetization. All volumes were aligned to the first picture of each run, coregistered with the anatomical data, and transformed to the Talairach coordinate space (Talairach and Tournoux 1988).

#### ROI analysis

**Figure 2:**
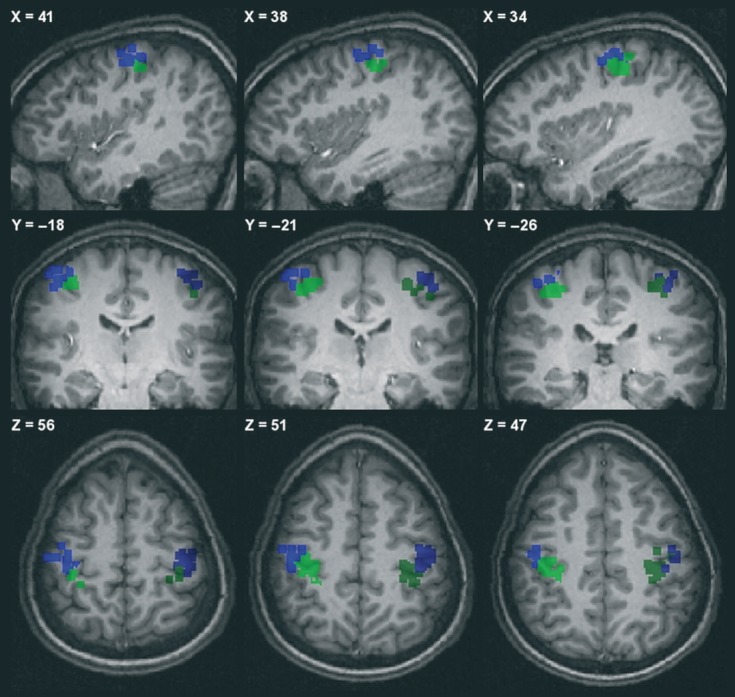
Subdivision of the primary sensorimotor cortex hand area in the more medial inferior and posterior (green) and more lateral superior and anterior (blue) part of all subjects. Left picture side corresponds to right hemisphere, right picture side to left hemisphere (coronal and transverse plane). *x*, *y*, *z*: Talairach coordinates.

As especially in the primary sensorimotor cortices intersubject anatomical variability is high (Woods 1996; White et al. 1997a; Rademacher et al. 2001), we chose a combined functional and anatomical approach to define our ROIs. Despite this intersubject anatomical variability, there is no hint for a handedness-specific effect on brain anatomy in the primary sensorimotor cortex (White et al. 1997b; Good et al. 2001). The central sulcus and the characteristic hand knob (Yousry et al. 1997) were used in all subjects for anatomical identification of the hand area of each subject separately. Then, for each subject, a whole-brain analysis of the localizer data with the significance threshold set to *q*(FDR) = 0.05 was performed in order to identify the functional relevant voxels on the individual level. Left hemisphere hand areas were assigned with the one-hand right finger movement against rest, and right hemisphere hand areas were assigned with one-hand left finger movement against rest. As it is known that there are at least two distinct hand representations within the primary motor cortex (Geyer et al. 1996), we divided the active regions within the hand knob in a more medial, inferior, and posterior part (representing 4p) close to area 3 in the depth of the central sulcus, and a more lateral, superior, and anterior part closer to area 6 (representing 4a). Within the two parts, ROIs were defined as the 125 voxels (5 × 5 × 5) around the most active voxel (Fig. 2). Due to the proximity of the primary motor and the primary sensory cortex, we cannot exclude that some of the measured fMRI activity originated from the primary sensory cortex. Hence, we refer to this region as primary sensorimotor cortex.

Coding was as follows: for right-handers, right hand was coded as dominant hand and left hand was coded as nondominant hand, whereas left hemisphere was coded as dominant hemisphere and right hemisphere was coded as nondominant hemisphere. For left-handers it was the other way around, left hand was coded as dominant hand and right hand was coded as nondominant hand, whereas right hemisphere was coded as dominant hemisphere and left hemisphere was coded as nondominant hemisphere.

For each subject, the time course of the BOLD signal of the 11 experimental conditions was extracted separately for the four ROIs (dominant and nondominant hand area divided in two subregions each). The time courses were averaged over the four blocks of each experimental condition and over all voxels of the respective ROI. The mean signal of the 2-sec preceding the finger movements was assigned as baseline and the percentage of signal change to baseline was extracted and averaged from second six to 18 of every block for each experimental condition and ROI separately. Data were analyzed separately for both handedness groups with four mixed models, namely one for moving finger of the dominant hand, one for the nondominant hand, one for both hands under undivided concentration, and one for both hands under divided concentration. In all analyses of the functional data, the random effect was subject and the fixed effects were hemisphere, subregion, attention level, and the interaction terms between the fixed effects. The fixed effects of the full models were tested with *F*-tests. The post hoc tests comparing two subconditions only were done with *t*-tests. In the case of missing data from an experimental condition (due to technical issues), we excluded subjects from the subanalysis (right-handers nondominant hand, *n* = 1; both hand undivided attention, *n* = 2; left-handers both hands undivided attention, *n* = 2).

Mixed-model calculations for the ROI analyses were performed with the nlme package (Pinheiro et al. 2012) in R 2.14.0 (R Development Core Team 2011). Reported significance levels were corrected for eight independent tests, to correct for the four models calculated in both handedness groups.

#### Whole-brain analysis

In order to investigate the effects of the different attention instructions on the whole-brain level, we calculated two mixed models separately for right- and left-handers. Random effect was always subject. The first analysis included the two fixed effects attention (attention-modulation-free condition, distraction, concentration) and motor task (both hands, dominant hand, nondominant hand), which were tested with *F*-tests. In the case of a significant attention effect, post hoc tests were performed with *t*-tests comparing distraction versus attention-modulation-free condition and concentration versus attention-modulation-free condition. For the post hoc tests, we were interested in the task-positive as well as the task-negative effects. Therefore, we analyzed not only the attention-related increase in activation expected in the dorsal attention network but also the decrease in activation expected in the ventral default network. The second random-effect analysis included the fixed effect divided concentration (concentration on dominant or nondominant hand while moving both index fingers), which was tested with *t*-tests. Data were normalized using the percent signal change transformation in Brainvoyager. For both handedness groups, *P*-value thresholds were set to <0.001 and minimum cluster sizes were set to 50 voxel. By using a threshold of <0.001 instead of a more stringent Bonferroni correction, we account for the smaller sample size and therefore less power of the left-hander group. In the case of missing data from an experimental condition, we excluded subjects from the whole-brain analysis (right-hander, *n* = 2; left-hander, *n* = 1).

#### Behavioral data analysis

Behavioral data, namely main tapping frequency ascertained by fast Fourier transformation of the time series of button presses (frequency with the highest amplitude between 0.5 and 3.5 Hz) and mean standard deviation of the tapping event in relation to the occurrence of the sound, were analyzed with the same four mixed models used for the ROI analyses. In all analyses of the behavioral data, subject was the random effect. For one-hand movements, fixed effect was attention type, whereas for bimanual movements, fixed effects were moving finger and attention type and the interaction term between moving finger and attention type. The fixed effects of the full models were tested with *F*-tests. In the case of missing data from an experimental condition, we excluded subjects from the subanalysis (right-hander nondominant hand, *n* = 1; dominant hand, *n* = 1; both hand undivided attention, *n* = 2; both hand divided attention, *n* = 1; left-hander nondominant hand, *n* = 1; dominant hand, *n* = 1; both hand undivided attention, *n* = 1).

Mixed-model calculations for the behavioral data analyses were performed with the nlme package (Pinheiro et al. 2012) in R 2.14.0 (R Development Core Team 2011). Reported significance levels are corrected for eight independent tests. As the behavioral data served as a control variable, and the two parameters of task performance cannot be seen as independent tests, we corrected only for four models calculated in both handedness groups to be more sensitive also for subtle changes in task performance.

## Results

### Behavioral results

Only one behavioral effect was significant: In the case of divided concentration, right-handers showed an overall lower movement frequency when concentrating on the nondominant hand (*F*[1,51] = 11.9, *P* = 0.009). All other results were not significant (*P* > 0.25), that is our attention modulations did neither influence the tapping frequency nor its variance. Especially for the nondominant hand, there was no influence of attention modulation on task performance of tapping frequency (right-hander *F*[2,34] = 1.0, *P* = 1.0, left-hander *F*[2,12] = 1.3, *P* = 1.0) or the standard deviation of the tapping in relation to the sound (right-hander *F*[2,34] = 1.7, *P* = 1.0, left-hander *F*[2,12] = 0.7, *P* = 1.0). Hence, attention-related BOLD differences cannot be simply attributed to variations in movement parameters.

### ROI results

For right-handers, in all conditions, the more lateral part of the primary sensorimotor cortex was more active than the more medial part (main effect subregion *P* < 0.01), whereby this effect was more pronounced in the dominant hemisphere when the finger of the dominant hand was moved (interaction hemisphere × subregion *F*[1,198] = 11.8, *P* = 0.006). The same main effect of subregion became significant for left-handers only when both fingers moved under undivided attention (*F*[1,66] = 9.6, *P* = 0.022) or (with a trend) when attention was divided (*F*[1,49] = 7.1, *P* = 0.083). No differences related to the experimental manipulations were observed between the suspected homologs of areas 4a and 4p (interaction attention level × subregion). Furthermore, there were no significant two- or three-way interactions (all *P* > 0.35). For the one-hand movements, activity strongly differed between the hemispheres in all analysis (all *P* < 5.0 × 10^−15^), reflecting higher activity in the hemisphere contralateral to the moving hand.

**Figure 3:**
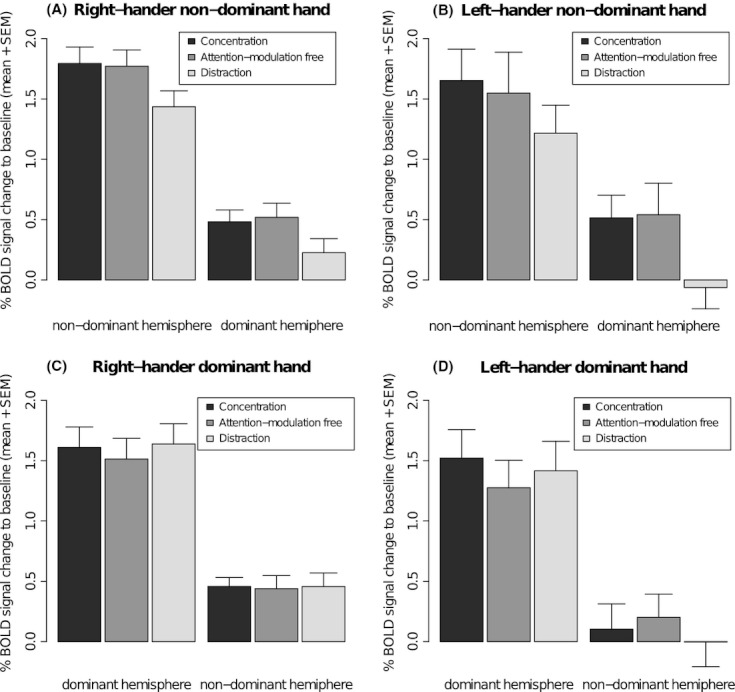
Effect of attention for the usage of the nondominant (A and B) and dominant (C and D) hand of right- (A and C) and left-handers (B and D). Distraction leads to a significant decrease of activation of the primary motor cortex of both hemispheres in both handedness groups for usage of the nondominant hand only. There are no significant interaction effects.

Our main finding regarding attentional modulation was an activity decrease in the primary sensorimotor cortex of both hemispheres under distraction when both handedness groups moved their nondominant hand (Fig. 3). This was true for both, right- and left-handers (main effect of attention right-handers *F*[2,187] = 11.0, *P* = 0.0003; left-handers *F*[2,77] = 8.9, *P* = 0.003).

Post hoc tests revealed no significant difference between concentration and attention-modulation-free conditions (right-hander *t*[123] = −0.1, *P* = 1.0; left-hander *t*[53] = 0.3, *P* = 1.0), but a decrease under distraction compared with attention-modulation-free blocks (right-handers *t*[123] = −4.0, *P* = 0.0009; left-handers *t*[53] = −3.6, *P* = 0.006) and with concentration blocks (right-handers *t*[123] = −4.3, *P* = 0.0003; left-handers *t*[53] = −4.3, *P* = 0.0006). No other effects of attention type became significant (all *P* > 0.77).

### Whole-brain results

**Figure 4:**
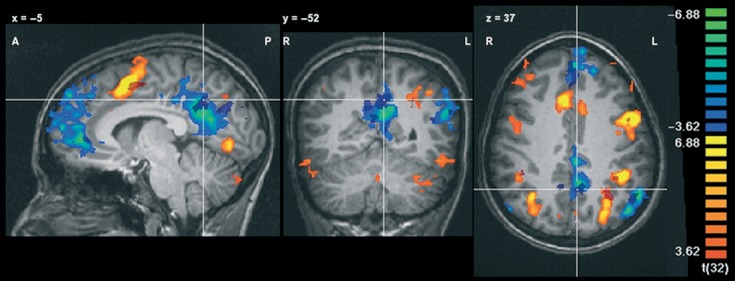
The activation map of the right-handers for the contrast distraction versus attention-modulation free. Blue and green colors depict deactivation under distraction, whereas red and yellow colors depict higher activation under distraction in comparison with attention-modulation-free condition. Dark red and dark blue are the same contrast for left-handers superimposed on the right-handers activation map. For both handedness groups, we set *P*-values to *P* < 0.001 and minimum cluster sizes to larger than 50 voxel. *x*, *y*, *z*: Talairach coordinates; R, L: right and left; A, P: anterior and posterior.

Attention-related task instructions affected neuronal activity in multiple brain regions including premotor areas, supplementary motor area (SMA), prefrontal regions, and parietal regions with a pronunciation on the left side (for the results of the *F*-tests for right- and left-handers, see Tables S1 and S2). Post hoc we compared the attention-modulation-free condition with distraction and concentration separately with *t*-tests. Reported are the most significant results of the right-hander group. Distraction led to lower activity in medial frontal (22.466 voxel, *P*_min_ = 2.0 × 10^−10^), medial posterior (13.554 voxel, *P*_min_ = 3.2 × 10^−9^), and left parieto-temporal cortex (7056 voxel, *P*_min_ = 2.9 × 10^−9^) in comparison with the attention-modulation-free condition. Activity in the dual task/distraction situation was higher in bilateral secondary motor areas (left hemisphere 8862 voxel, *P*_min_ = 2.1 × 10^−12^, right hemisphere 4223 voxel, *P*_min_ = 8.1 × 10^−9^) and medial motor areas (10.148 voxel, *P*_min_ = 2.7 × 10^−13^) as well as in a bilateral parietal network (left hemisphere 8055 voxel, *P*_min_ = 1.4 × 10^−12^; right hemisphere 7730 voxel, *P*_min_ = 4.8 × 10^−11^). The left-hander group showed smaller but overlapping clusters in comparison to the right-hander group (Fig. 4).

**Figure 5:**
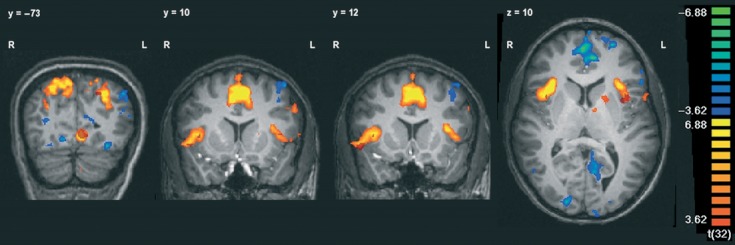
The activation map of the right-handers for the contrast distraction versus attention-modulation free. Blue and green colors depict deactivation under distraction, whereas red and yellow colors depict higher activation under distraction in comparison with attention-modulation-free condition. Superimposed in dark red are the significant spots in right-handers for the contrast concentration versus attention-modulation-free condition. In both analyses, we set *P*-values to *P* < 0.001 and minimum cluster sizes to larger than 50 voxel. *y*, *z*: Talairach coordinates; R, L: right and left.

The comparison concentration versus attention-modulation-free trials revealed some small activity spots in the right inferior frontal gyrus (158 voxel, *P*_min_ = 5.0 × 10^−6^), bilateral insula (left hemisphere 135 voxel, *P*_min_ = 6.0 × 10^−6^; right hemisphere 67 voxel, *P*_min_ = 4.1 × 10^−5^), left-parietal (54 voxel, *P*_min_ = 3.9 × 10^−5^), and left occipital (extrastriatal visual) cortex (405 voxel, *P*_min_ = 8.8 × 10^−7^) only in the right-hander group. All these spots displayed higher activity under concentration. They correspond to regions also found to be more active in the distraction versus attention-modulation-free contrast of the right-hander (Fig. 5). The divided concentration conditions did not show any significant voxels in both left- and right-handers.

## Discussion

This study found an influence of attention on activity in the primary sensorimotor cortex of both hemispheres when (a) left- or right-handers moved their nondominant hand and (b) subjects were distracted by an attention-demanding second (dual) task. In the latter case, activity in primary sensorimotor cortex was reduced compared with attention-modulation-free and concentration trials. The reduction of activity in primary sensorimotor cortex activity was not limited to the contralateral hemisphere or to the subregion 4a within the primary sensorimotor cortex, but was observed in both hemispheres and both subregions to the same extent. The other attention-related experimental condition, namely concentration on the moving finger(s), had no effect on primary sensorimotor cortex activity. Also we did not find an influence of attention on unimanual movements of the dominant hand or on bimanual movements. Moreover, with the exception of the condition in which right-handers had to pay attention to the nondominant hand during bimanual movements, our attention-related experimental modulations had no impact on behavioral performance. This is an important finding, as otherwise the observed distraction-driven fMRI effects in the primary sensorimotor cortex could have been attributed to say differences in tapping frequencies. As no behavioral alterations were observed, the reduced activity in primary motor cortex under distraction very likely reflects top-down modulation by higher cortical areas.

The whole-brain analyses confirm that the distraction condition was able to modulate activity not only in the primary sensorimotor cortex but also in a large variety of brain regions including higher motor areas. These areas are known to be part of the (dorsal) frontoparietal attention network (Collette et al. 2005; Fox et al. 2005; Nebel et al. 2005). At the same time, activity in a network resembling the default or resting state network (Fox et al. 2005) was found to be suppressed during distraction, a finding observed in tasks with higher difficulty (McKiernan et al. 2003). Together, these findings support the idea that the dual task demanded attentional resources that were withdrawn from the motor task. The concentration instruction led to higher activity in some small spots, all of which correspond to regions that also showed higher activity under distraction (right inferior frontal gyrus, bilateral insula, left-parietal cortex, and occipital cortex). This observation is consistent with results of Nebel et al. (2005), who showed that focused, for example, concentration on one task, and divided attention, for example, performing two tasks simultaneously, depend on overlapping networks. There were no detectable effects of the concentration conditions on the default or resting state network. Possible reasons for the rather weak impact of our concentration in comparison to our distraction instruction are given below.

### Effect of distraction on primary sensorimotor cortex activity

With our very simple tapping task, we observed an influence of distraction when the nondominant, but not when the dominant index finger had to be moved in both handedness groups. This result is comparable with that of Johansen-Berg and Matthews (2002), where the authors likewise found a decrease of activity under distraction for right-handers moving their left hand. In their study, the influence of distraction was limited to the primary sensorimotor cortex of the contralateral hemisphere, whereas in our study the effect was seen in the primary sensorimotor cortex of both hemispheres. The main difference between the studies is ROI definition. Johansen-Berg and Matthews (2002) chose a solely anatomical definition, whereas we defined the ROIs combining anatomical and functional information for each subject separately. However, with their whole-brain group analysis, Johansen-Berg and Matthews (2002) could identify a spot in the ipsilateral hemisphere in the sulcus centralis, which also showed a decrease of activity but which was not included in their anatomically defined ROI. Hence, there is evidence for a bihemispheric effect in their study as well.

Findings regarding primary sensorimotor cortex activity in the ipsilateral hemisphere per se are relatively heterogeneous. For example, some (Wassermann et al. 1991, 1994; Cramer et al. 1999) but not all (Jäncke et al. 1998; Nirkko et al. 2001) studies showed an ipsilateral coactivation during motor tasks. There are also hints that ipsilateral active regions lie more lateral in comparison with contralateral activity (Wassermann et al. 1994), and that active regions can change with motor learning (Sanes et al. 1992). In our study, we observed a slight coactivation in the ipsilateral finger area in the primary sensorimotor cortex, which was also affected by the distraction condition.

Rodríguez et al. (2004) reported a decrease of activity within contralateral primary motor cortex under distraction while subjects performed a phasic movement (increasing the metacarpusphalange joint angle from 0° to 45° while stretching an elastic band and passively returning to the initial position) with the dominant hand. Using a voxel-based fine-mapping approach and a time course analysis, they showed a significant decrease of active area size and signal intensity within the contralateral primary motor cortex. Furthermore, they could show a reconfiguration of the active field in the contralateral primary motor cortex whereby some voxels were active solely under the basal condition while others were active under distraction. It is important to note that before starting fMRI, Rodríguez et al. (2004) made sure to include only subjects who were able to perform the task correctly. However, they did not check for behavioral differences in the fMRI experiment itself. Thus, confound from behavioral differences cannot be excluded in their study. Under the premise that there were no such behavioral differences in the fMRI task, the results of Rodríguez et al. (2004) demonstrate that with a more complex motor task together with a fine-mapping analysis approach influences of attention on the primary motor cortex can be observed while the dominant hand is used as well.

In our study, distraction entailed activity reductions in the primary motor cortex only when both left- and right-handers made a unimanual movement with their nondominant hand. Distraction had no effect on primary motor cortex activity when the dominant hand moved. This finding indicates that distraction by a demanding cognitive task drains resources in the sense of a push/pull mechanism from primary motor cortex only when the neuronal representation of the movement is less efficient as it is the case with the less well-trained, nondominant hand. Simple, externally paced finger tapping with the dominant hand, on the other side, can be considered such an overlearned, heavily trained task that even performing a cognitive task simultaneously does not compromise its very efficient representation in the activated primary motor cortex network, although the dual task per se activates additional higher motor areas. In this respect, it is noteworthy that in everyday life, one can often observe persons who make rhythmic movements with their hands (e.g., tapping on the desk, playing with a pen) when engaged in demanding cognitive tasks. It is easily conceivable that with a less well-trained and internally paced motor task, like making U-type movements (Binkofski et al. 2002) activity changes in primary motor cortex during distraction could have been observed with the dominant hand as well. The finding that the activity reduction in the nondominant motor cortex did not affect behavioral performance in our view again is attributable to the fact that a very simple task was performed. With a more demanding, less well-trained task the activity reduction likely would have been accompanied with behavioral deficits. Hence, we propose that whether attention-related modulation of the primary motor cortex activity occurs depends on the routine and complexity of the motor task.

### Differentiation between 4a and 4p

In this study, no differences in attention-dependent neuronal activation emerged between the more medial, posterior, and inferior finger area, presumably representing area 4p, and the more lateral, anterior, and superior part of the finger area, presumably representing area 4a. Previous studies which observed such differences (Johansen-Berg and Matthews 2002; Binkofski et al. 2002) defined 4a and 4p anatomically for their ROI analysis, whereas we divided the functionally identified active finger area in the more medial part close to area 3 and the more lateral part close to area 6. Binkofski et al. (2002) verified their anatomical definition of regions with probabilistic maps of postmortem brains and could demonstrate a clear linear relationship between motor attention and neuronal activity exclusively in 4p of the contralateral hemisphere. Johansen-Berg and Matthews (2002) chose an anatomically less strict definition, and observed not only a significant effect in 4p but also – at least a nominal significant– decrease of activation in 4a of the contralateral hemisphere. With our functional definition of two distinct parts within the anatomically identified primary sensorimotor hand area, we were not able to verify subregional differences regarding attentional modulation. Instead, we could demonstrate an effect of distraction not only on the contralateral but also on the ipsilateral hemisphere, more precisely in the finger area of the opposite index finger.

### Concentration instruction

We observed no neuronal activity changes in primary sensorimotor cortex during the concentration instruction, no matter of whether concentration was divided or undivided, with respect to the attention-modulation-free condition. Corresponding to this result, on the whole-brain level, we found only some small spots that were more active in the undivided concentration condition. All of them were identical to those regions, which were more active under distraction in comparison with attention-modulation free, including the largest cluster located in the extrastriatal visual cortex of the left hemisphere. A possible explanation for this effect is that in both conditions, attention was directed to the visual input (number presented on the screen in the distraction task, moving finger in the concentration task), a process known to enhance activity in visual cortex through top-down modulation (e.g., Hopfinger et al. 2000; Müller et al. 2003).

Unlike us, Binkofski et al. (2002) could show that concentration on motor action (right-handers dominant hand) can increase activity specifically in area 4p of the contralateral hemisphere. They manipulated attention in three steps: attention to the moving finger, attention to a computer screen without further task, and attention to the screen while counting flashes on the screen. They also required a more complex and less common U-shaped movement with the right hand. Apart from the fact that their subjects had to perform a more complex motor task, the reason for the varying results may relate to the specific concentration instruction. Indeed, there are plenty of different concentration instructions, as for example, internal versus external focus (Wulf and Prinz 2001; Zentgraf et al. 2009) or concentration on the action itself versus on the intention to make a movement (Jueptner et al. 1997; Lau et al. 2004). The present results suggest that an instruction, which intended to just shift attention to a finger while performing a very simple movement, is not able to alter brain activity profoundly. Hence, effects of concentration on motor and other brain areas may be limited to situations where (a) concentration is devoted to an external rather than internal focus and/or (b) a more complex, not highly overlearned, movement is required.

## Conclusion

To sum up, we could show a decrease of activation in primary sensorimotor cortex in both the contra- and the ipsilateral hemisphere for right- and left-handers when they used their nondominant hand in an externally paced simple button press task and when they were distracted by a second, attention-demanding task. With this simple task, no effect for the dominant hand or for concentration instructions was seen in the primary motor cortex. Usage of dominant versus nondominant hand, complexity of both motor and attention task, and training status seem to be relevant factors that determine attention-related activity modulations in the primary sensorimotor cortex.

## Supporting Information

**Table S1.** The results of the whole-brain fMRI analysis for right-handers main effect of attention.

**Table S2.** The results of the whole-brain fMRI analysis for left-handers main effect of attention.
