# Supplementary material for: Attention-dependent modulation of neural activity in primary sensorimotor cortex
Source: Brain Behav. 2013 Jan 4;3(2):54–66. doi: 10.1002/brb3.114 (PMC3607147; doi:10.1002/brb3.114)
Supplement: Supplementary file 1 [file brb30003-0054-SD1.docx]

Supplemental Material for

“Attention-dependent modulation of neural activity in primary sensorimotor cortex”

Annette Milnik,^1,2^ Isabella Nowak,^2^ and Notger G. Müller^1,2,3^

**Authors Affiliations:**

1 Department of Neurology, University of Magdeburg, Magdeburg, Germany

2 Cognitive Neurology Unit & Brain Imaging Center, Clinic for Neurology, Johann Wolfgang Goethe-University, Frankfurt, Germany

3 German Center for Neurodegenerative Diseases, Magdeburg, Germany

SUPPLEMENTARY TABLE I. Shown are the results of the whole-brain fMRI analysis for right-handers main effect of attention. Information of the region, Talairach-coordinates and test statistics are reported for the most significant voxel of the whole cluster. BA: Brodmann area; Hem: Hemisphere (L: Left; R: Right; B: Both/Medial); X, Y, Z: Talairach-coordinates.

| Region | BA | Hem | X | Y | Z | N | *F*_(2,32)_ | p |
| --- | --- | --- | --- | --- | --- | --- | --- | --- |
| **Frontal lobe** |  |  |  |  |  |  |  |  |
| Precentral Gyrus | 4 | L | -51 | -4 | 46 | 386 | 16.86 | 1.00E-05 |
| Middle Frontal Gyrus | 6 | L | -24 | -4 | 49 | 641 | 18.37 | 5.00E-06 |
| Medial Frontal Gyrus | 6 | B/L | -6 | 5 | 52 | 3096 | 25.58 | 2.31E-07 |
| Middle Frontal Gyrus | 6 | R | 27 | 2 | 49 | 505 | 15.18 | 2.30E-05 |
| Middle Frontal Gyrus | 8 | L | -39 | 17 | 43 | 236 | 19.62 | 3.00E-06 |
| Middle Frontal Gyrus | 8 | L | -27 | 20 | 46 | 1312 | 22.33 | 8.49E-07 |
| Inferior Frontal Gyrus | 9 | L | -42 | 2 | 31 | 1202 | 20.83 | 2.00E-06 |
| Inferior Frontal Gyrus | 9 | L | -42 | 2 | 30 | 1202 | 20.25 | 2.00E-06 |
| Medial Frontal Gyrus | 9 | L | -9 | 44 | 22 | 53 | 14.75 | 2.90E-05 |
| Superior Frontal Gyrus | 9 | R | 42 | 35 | 28 | 82 | 11.80 | 0.000145 |
| Middle Frontal Gyrus | 10 | L | -27 | 56 | 10 | 67 | 15.85 | 1.60E-05 |
| Medial Frontal Gyrus | 10 | B | 3 | 53 | 13 | 7327 | 33.15 | 1.59E-08 |
| Paracentral Lobule | 31 | R | 12 | -13 | 46 | 52 | 16.65 | 1.10E-05 |
| Inferior Frontal Gyrus | 46 | L | -51 | 26 | 13 | 202 | 15.38 | 2.10E-05 |
| Inferior Frontal Gyrus | 47 | R | 36 | 29 | -8 | 88 | 17.69 | 7.00E-06 |
| **Parietal lobe** |  |  |  |  |  |  |  |  |
| Postcentral Gyrus | 2 | L | -63 | -22 | 31 | 155 | 17.23 | 8.00E-06 |
| Postcentral Gyrus | 2 | R | 57 | -19 | 31 | 816 | 21.52 | 1.00E-06 |
| Precuneus | 7 | L | -15 | -46 | 55 | 50 | 21.04 | 1.00E-06 |
| Superior Parietal Lobule | 7 | R | 12 | -70 | 55 | 60 | 15.98 | 1.50E-05 |
| Precuneus | 7 | R | 15 | -76 | 43 | 286 | 15.85 | 1.60E-05 |
| Precuneus | 19 | L | -27 | -70 | 40 | 1101 | 25.80 | 2.12E-07 |
| Angular Gyrus | 39 | R | 30 | -58 | 31 | 386 | 16.07 | 1.50E-05 |
| Inferior Parietal Lobule | 40 | L | -42 | -43 | 40 | 768 | 27.21 | 1.25E-07 |
| **Temporal lobe** |  |  |  |  |  |  |  |  |
| Inferior Temporal Gyrus | 20 | L | -51 | -55 | -11 | 285 | 18.57 | 4.00E-06 |
| Inferior Temporal Gyrus | 20 | R | 54 | -49 | -14 | 193 | 14.05 | 4.20E-05 |
| Inferior Temporal Gyrus | 21 | L | -57 | -13 | -14 | 75 | 12.66 | 8.90E-05 |
| Middle Temporal Gyrus | 21 | L | -54 | -22 | -5 | 166 | 16.72 | 1.10E-05 |
| Middle Temporal Gyrus | 21 | L | -54 | -1 | -17 | 103 | 12.98 | 7.50E-05 |
| Superior Temporal Gyrus | 22 | R | 51 | -7 | -8 | 143 | 15.51 | 2.00E-05 |
| Superior Temporal Gyrus | 39 | L | -48 | -61 | 19 | 4368 | 34.01 | 1.21E-08 |
| Superior Temporal Gyrus | 39 | L | -33 | -55 | 31 | 103 | 11.65 | 0.000158 |
| Middle Temporal Gyrus | 39 | R | 45 | -58 | 13 | 186 | 21.89 | 1.00E-06 |
| Superior Temporal Gyrus | 41 | L | -45 | -37 | 7 | 121 | 12.76 | 8.40E-05 |
| **Occipital lobe** |  |  |  |  |  |  |  |  |
| Lingual Gyrus | 18 | L | -30 | -70 | -5 | 104 | 12.71 | 8.70E-05 |
| Lingual Gyrus | 18 | L | -15 | -85 | -8 | 194 | 13.92 | 4.50E-05 |
| Cuneus |  | L | -18 | -94 | 1 | 154 | 15.70 | 1.80E-05 |
| **Limbic lobe / sub lobar** |  |  |  |  |  |  |  |  |
| Cingulate Gyrus | 31 | B | -6 | -28 | 37 | 5623 | 25.90 | 2.04E-07 |
| Parahippocampal Gyrus | 36 | L | -30 | -31 | -11 | 465 | 25.69 | 2.22E-07 |
| Amygdala |  | L | -21 | -10 | -14 | 93 | 17.78 | 6.00E-06 |
| Amygdala |  | R | 24 | -1 | -14 | 112 | 13.13 | 6.80E-05 |
| Hippocampus |  | L | -30 | -10 | -14 | 226 | 20.77 | 2.00E-06 |
| Insula | 13 | L | -42 | -25 | 19 | 64 | 12.62 | 9.10E-05 |
| Insula | 13 | L | -39 | -10 | 13 | 69 | 10.96 | 0.000236 |
| Insula | 13 | L | -30 | 17 | 16 | 387 | 25.06 | 2.82E-07 |
| Insula | 13 | R | 39 | -4 | 16 | 206 | 21.26 | 1.00E-06 |
| Claustrum |  | R | 30 | 17 | 10 | 732 | 18.01 | 6.00E-06 |

SUPPLEMENTARY TABLE II. Shown are the results of the whole-brain fMRI analysis for left-handers main effect of attention. Information of the region, Talairach-coordinates and test statistics are reported for the most significant voxel of the whole cluster. BA: Brodmann area; Hem: Hemisphere (L: Left; R: Right; B: Both/Medial); X, Y, Z: Talairach-coordinates.

| Region | BA | Hem | X | Y | Z | N | *F*_(2,12)_ | p |
| --- | --- | --- | --- | --- | --- | --- | --- | --- |
| **Frontal lobe** |  |  |  |  |  |  |  |  |
| Medial Frontal Gyrus | 10 | L | -3 | 56 | 10 | 51 | 30.31 | 2.00E-05 |
| **Parietal lobe** |  |  |  |  |  |  |  |  |
| Precuneus | 7 | L | -6 | -52 | 34 | 246 | 28.01 | 3.00E-05 |
| Precuneus | 7 | R | 21 | -52 | 55 | 118 | 24.09 | 6.30E-05 |
| Inferior Parietal Lobule | 40 | R | 54 | -28 | 22 | 55 | 22.70 | 8.30E-05 |
| Postcentral Gyrus | 43 | R | 51 | -19 | 16 | 288 | 36.86 | 8.00E-06 |
| **Temporal lobe** |  |  |  |  |  |  |  |  |
| Middle Temporal Gyrus | 21 | R | 54 | -55 | 4 | 82 | 42.46 | 4.00E-06 |
| Middle Temporal Gyrus | 21 | R | 57 | -13 | -8 | 157 | 29.87 | 2.20E-05 |
| Superior Temporal Gyrus | 22 | R | 45 | -55 | 16 | 113 | 24.00 | 6.40E-05 |
| Superior Temporal Gyrus | 38 | R | 45 | 8 | -17 | 50 | 36.28 | 8.00E-06 |
| Superior Temporal Gyrus | 39 | L | -51 | -61 | 19 | 351 | 27.07 | 3.60E-05 |
| Superior Temporal Gyrus | 39 | R | 54 | -55 | 22 | 520 | 57.59 | 7.06E-07 |
| **Limbic lobe** |  |  |  |  |  |  |  |  |
| Posterior Cingulate | 23 | R | 3 | -46 | 22 | 223 | 44.55 | 3.00E-06 |
| Parahippocampal Gyrus | 28 | L | -24 | -19 | -14 | 65 | 29.54 | 2.30E-05 |
| Cingulate Gyrus | 31 | L | -12 | -37 | 34 | 127 | 26.38 | 4.00E-05 |
| Anterior Cingulate | 32 | L | -3 | 32 | -5 | 50 | 28.32 | 2.90E-05 |
